# Computing generalized cophenetic distances under all *L*_*p*_ norms: A near-linear time algorithmic framework

**DOI:** 10.1371/journal.pcbi.1013069

**Published:** 2025-06-10

**Authors:** Paweł Górecki, Alexey Markin, Sriram Vijendran, Oliver Eulenstein

**Affiliations:** 1 Faculty of Mathematics, Informatics and Mechanics, University of Warsaw, Warsaw, Mazowieckie, Poland; 2 Virus and Prion Research Unit, National Animal Disease Center, USDA-ARS, Ames, Iowa, United States of America; 3 Department of Computer Science, Iowa State University, Ames, Iowa, United States of America; Ecole Normale Superieure, FRANCE

## Abstract

The cophenetic distance is a well-established metric in biology used to compare pairs of trees represented in a vector format. This distance was introduced by Cardona and his co-authors, building on the foundational work of Sokal and Rohlf, which dates back over 60 years. It is widely recognized for its versatility since it can analyze trees with edge weights using various vector norms. However, when comparing large-scale trees, the quadratic runtime of the current best-known (i.e., naïve) algorithm for computing the cophenetic distance can become prohibitive. Recently, a new algorithmic framework with near-linear time complexity has been developed to calculate the distances of a generalized class of cophenetic distances, which are derived from the work of Sokal and Rohlf. This improvement not only allows the cophenetic distance to be utilized in large-scale studies but also enhances the versatility of these studies by incorporating generalized variants of the cophenetic distance. However, the framework is limited to applying only the *L*_1_ and *L*_2_ vector norms, which significantly restricts the versatility of generalized cophenetic distances in large-scale applications. To address this limitation, we present a near-linear time algorithmic framework for computing the generalized cophenetic distances across all *L*_*p*_ vector norms. In our scalability study, we showcase the practical performance of our unrestricted algorithmic framework. Furthermore, we investigate the applicability of the generalized cophenetic distances by analyzing the distributions of key components of these distances under various vector norms.

## Author summary

Biological research often relies on large-scale comparisons of evolutionary trees to extract valuable insights across different subfields of biology. To effectively compare trees on a large scale, sensitive metrics are needed to assess differences in topology and branch lengths alongside efficient computational algorithms. In this study, we focus on the classic cophenetic distance, a metric that compares pairs of trees represented in vector format. The cophenetic distance can analyze trees with edge weights using various vector norms, making it highly versatile. Recently, a fast algorithm was developed to calculate generalized cophenetic distances, including the cophenetic distance itself, which has enabled large-scale studies and expanded the types of cophenetic distances applicable for tree pair comparisons. However, this algorithm is limited to *L*_1_ and *L*_2_ norms, which greatly reduces the versatility of the cophenetic distance. To overcome this limitation, we introduce a fast algorithm that computes the generalized cophenetic distance for all vector norms, making it suitable for large-scale studies. We also conduct a scalability study to demonstrate the effectiveness of our algorithm in practice, and we analyze the distributions of key representatives of cophenetic distances across various vector norms.

## Introduction

The cophenetic distance, introduced by Cardona *et al*. [1], originates from the pioneering work of Sokal and Rohlf over 60 years ago [2] and has gained substantial recognition in biology due to its reputation for reliability in analysis. This distance is a vector-based metric that is more versatile than many other commonly used tree metrics [3–5], as it can be applied to tree pairs with edge weights and analyzed using various vector norms. This capability allows for a more in-depth analysis, particularly when comparing similar tree topologies. Consequently, the cophenetic distance has broad applicability across various fields, such as phylogenetics [6–8], genomics [9], ecology [10–12], epidemiology [13,14], and conservation biology [15].

**Fig 1:**
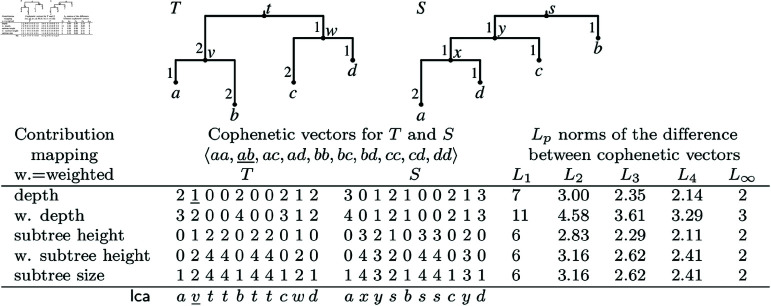
Cophenetic distances under various *L*_*p*_ norms and contribution functions. The vectors have values computed for the least common ancestor (lca, see the bottom row) of the corresponding taxon pairs. For example, ${\text{lca}}_{T}(a,b)$ is *v*, and the second position in cophenetic vectors for *T* reflects the contribution of *v*, that is, the depth of *v* is 1.

The cophenetic distance between a pair of rooted binary trees is defined based on their representation as cophenetic vectors. A *cophenetic vector* for a rooted tree assigns a value to each pair of taxa within the tree, representing the depth of their least common ancestor in that particular tree. Cophenetic vectors encode their corresponding trees equivalently [1], allowing the distance between a pair of trees to be measured as a *L*_*p*_ norm of the vector difference of the corresponding trees. Vector norms are beneficial not only for biological data analysis [16] but also applicable in various fields, such as statistical analyses [17] and machine learning [18]. An example of various vector norms is depicted in Fig 1.

With the advent of genome-scale data, the established reputation of the cophenetic distance and its analysis using various norms has led to increased interest among researchers in utilizing this metric for large-scale biological analyses. Such analyses include phylogenetics [19], median tree estimation [20,21], comparison of evolutionary process models [22], balancing of trees and networks [6,7], studies on theoretical properties of cophenetic $L_{\infty }$ norm [23], and representative sampling [10]. Calculating cophenetic distances using the naïve algorithm, which has a quadratic time complexity, is slow and impractical for large datasets. Fortunately, an efficient algorithmic framework now enables near-linear time computations for a *generalized class of cophenetic distances* for the *L*_1_ and *L*_2_ norms [24]. This advancement makes it feasible to conduct large-scale analyses. However, despite these advancements, algorithms for calculating cophenetic distances in sub-quadratic time for other *L*_*p*_ norms are still unknown.

In this paper, we present a near-linear time algorithmic framework for calculating the *L*_*p*_ norms for the generalized class of cophenetic distances. We demonstrate the performance of our algorithmic framework through a scalability study. Furthermore, we analyze the distributions of key representatives from the generalized class of cophenetic distances across various *L*_*p*_ norms. An implementation of the algorithmic framework can be found on GitHub [25].

**Related work.** The cophenetic distance is frequently used in phylogenetics to measure the similarity between two trees; both trees are encoded as vectors and are then compared in the corresponding vector space using different norms, such as the *L*_1_, *L*_2_, or $L_{\infty }$ norm. Another popular metric that utilizes vector encodings is the path-difference distance, and both metrics can be naïvely computed in *O*(*pn*^2^) time for a pair of trees with *n* taxa under the *L*_*p*_ norm. This quadratic runtime is highly restrictive in the realm of large-scale phylogenetic analysis. [26,27] addressed the quadratic barrier for the path-difference metric under the *L*_*p*_ norm by proposing near-linear time algorithms. For the cophenetic distances, [24] proposed a novel algorithmic framework to efficiently compute the distance between a pair of trees under the *L*_1_ and *L*_2_ norms in $O(n\log^{2}\;n)$ time and O(nlogn) time, respectively [24]. Furthermore, [24] demonstrated that the framework can be applied to a broad class of cophenetic metrics constructed using path-monotonic mappings, which are monotonic on the paths of the input trees. For instance, the original cophenetic distance is derived from the depth of a node, which is a path-monotonic mapping. This condition enables the development of hybrid approaches, resulting in new cophenetic costs rather than metrics, by allowing distinct path-monotonic mappings for each input tree. Such an approach may be particularly suitable when the input trees originate from different sources.

**Contribution.** We introduce a near-linear time algorithmic framework for computing the *L*_*p*_ norms of a broad class of cophenetic distances. Our framework achieves the following time complexities: O(pnlogn) for even values of *p*, $O(n\log^{2}\;n\;+\;pn\log n)$ for odd values of *p*, and $O(n\log^{2}\;n)$ under the $L_{\infty }$ norm.

Our approach begins by partitioning the input trees into four approximately equal-sized subtrees. Based on the placement of taxon pairs within these subtrees, we identify several categories of taxon pair locations. For each category, we develop a specific method to compute their contributions to the cophenetic distance. The first category requires four recursive calls on controlled (smaller) subtrees extracted from the input trees. Other categories can be computed in linear time. For the final category, the computation depends on the parity of *p*, resulting in time complexities of O(plogn) and *O*(*pn*) for odd and even *p*, respectively. By integrating these methods, we develop a unified divide-and-conquer algorithm for computing cophenetic distances under *L*_*p*_ norms with finite *p*. Furthermore, we extend this approach to efficiently compute the $L_{\infty }$ norm of the cophenetic distance.

We demonstrate the efficiency of our algorithmic frameworks through experimental evaluations. We analyze the runtime of the proposed algorithm for tree pairs with varying numbers of taxa in a scalability study under multiple *L*_*p*_ norms. Our findings indicate that our implementation of the divide-and-conquer strategy significantly outperforms the runtime of the best-known quadratic algorithm for pairs of trees with more than 400 taxa across all tested norms p≤100.

Lastly, we investigated various types of cophenetic distances under different *L*_*p*_ norms, treating them as distributions of pairwise distances. The trees for each distribution were randomly generated using the uniform model and the Yule model [28]. Our analyses reveal that the class of cophenetic distances provides a high degree of diversity under larger *L*_*p*_ norms, which can benefit numerous applications in comparative phylogenetics.

## Methods

This section presents algorithms for computing the *L*_*p*_ norms of cophenetic distances. We begin with the necessary definitions and outline the method for identifying a median vertex in a rooted tree. Next, we describe how input trees are partitioned into four approximately equal subtrees using median vertices. Based on the positions of taxon pairs within these subtrees, we classify them into several categories. For each category, we propose distinct algorithms to calculate their contributions to the cophenetic distance. Finally, we introduce a unified algorithm for computing cophenetic distances for all finite norms and the $L_{\infty }$ norm. We also analyze and present the time and space complexities of the proposed algorithms.

### Definitions

We introduce needed notation and terminology partially following [24]. Let $T=\langle V_{T},E_{T}\rangle$ be a rooted binary tree, and let *v* and *w* be vertices in *T*. The root of *T* is denoted by root(T). The least common ancestor of *v* and *w* in *T* is denoted by ${\text{lca}}_{T}(v,w)$. We use v⪯w to denote that *w* lies on the path between *v* and the root of *T*. Note that v≺w means v⪯w and v≠w. A vertex *v* is called *strictly internal* in *T*, if *v* is neither a leaf nor the root. For any non-root vertex *v*, v.parent and v.\textsf{sibling} denote the parent and sibling of *v*, respectively. The set of leaves in *T* is denoted by *L*_*T*_, and the number of leaves by |T|. Similarly, $L_{T}(v)$ denotes the set of all leaves reachable from *v*, and |v| represents the size of $L_{T}(v)$. A *weighted* tree *T* is a rooted binary tree with an edge weight function $w_{T}:E_{T}\to {\mathbb{R}}_{+}$.

We write that a function $\xi :V_{T}\to \mathbb{R}$ is *path-monotonic* if, for every *v* and *w* such that v⪯w, either ξ(v)≥ξ(w) (descending) or for every *v* and *w* such that v⪯w, ξ(v)≤ξ(w) (ascending). In this article, we distinguish the following three *contribution functions*, defined for unweighted trees, where for a vertex *v* from *T* its contribution is:

- the depth of a *v*, i.e., the number of edges on the path from *v* to the root of *T* [1],
- the height of the subtree rooted at *v*, defined as the maximum number of edges in any path from *v* to a leaf in the subtree,
- the number of leaves in the subtree of *T* rooted at *v*.

Note that the first contribution function is descending, whereas the other two are ascending.

A similar definition applies to weighted depth and height in weighted trees, but instead of counting the number of edges, we sum the weights of edges. See examples in Fig 1. Note that our definition allows for the contribution of the root. Therefore, a tree may also include an additional rooting edge (with an associated weight), whose bottom vertex is the root, if necessary.

Let *T* and $T^{\prime }$ be trees with the same set of leaves $L(T)=L(T^{\prime })=\{x_{1},x_{2},\ldots ,x_{n}\}$, where the ordering of leaves is fixed. Let $\xi :V_{T}\to \mathbb{R}$ and $\xi^{\prime }:V_{T^{\prime }}\to \mathbb{R}$ (both either ascending or descending) be two path-monotonic contribution functions.

The *cophenetic vector* of *T* is defined as $\phi (T,\xi ):=[\xi ({\text{lca}}_{T}(x_{i},x_{j})){]}_{i\leq j}$, and similarly for $T^{\prime }$. The *L*_*p*_*-cophenetic distance* with respect to the contribution functions ξ and $\xi^{\prime }$ is defined for 1≤p≤∞ as:


$$d_{p}(T,T^{\prime }):={\left\|\phi (T,\xi )-\phi (T^{\prime },\xi^{\prime })\right\|}_{p},$$

where $\|\ldots {\|}_{p}$ is the *L*_*p*_ norm. That is, *d*_*p*_ is the *L*_*p*_ norm of the difference between the two cophenetic vectors induced by ξ and $\xi^{\prime }$.

Formally, in our case, if *p* is finite,


$$d_{p}(T,T^{\prime })=(\sum_{1\leq i\leq j\leq n}|\xi ({\text{lca}}_{T}(x_{i},x_{j}))-\xi^{\prime }({\text{lca}}_{T^{\prime }}(x_{i},x_{j})){|}^{p}{)}^{\frac{1}{p}},$$

otherwise


$$d_{\infty }(T,T^{\prime })=\max_{i,j}|\xi ({\text{lca}}_{T}(x_{i},x_{j}))-\xi^{\prime }({\text{lca}}_{T^{\prime }}(x_{i},x_{j}))|.$$

It should be clear that *d*_*p*_ is a metric [1] as long as both ξ and $\xi^{\prime }$ are based on the same type of contribution functions (e.g., depth).

### Median vertex in a rooted tree

A vertex *t* of a rooted tree *T* divides the tree into two parts: the subtree of *T* rooted at *t*, denoted by *T*_*t*_ and referred to as the *lower tree* with respect to *t*, and the tree *T*^*t*^, called the *upper tree*, which is obtained by replacing *T*_*t*_ with a leaf. The vertex *t*, as introduced in the next lemma, is called a *median vertex* and can be computed in *O*(*n*) time.

**Lemma 1** (The existence of a median vertex; [24]). *For every rooted binary tree T of size* n≥2 *there is a vertex t such that* $\frac{n}{2}\leq |T^{t}|\leq \frac{3n}{4}+1$ *and* $\frac{n}{4}\leq |T_{t}|\leq \frac{n}{2}$.

We will present the proof for Lemma 1, as it was not included in [24].

*Proof:* Let *t* be a vertex computed as follows: initialize *t* as the root of *T*, and repeatedly update *t* to its child with the largest subtree size until $|t|\leq \frac{n}{2}$. We show that the resulting vertex *t* satisfies the condition.

Let $t^{\prime }$ be the sibling of *t* and *p* be the parent of *t*. We have |*T*_*t*_| = |*t*|, $|t^{\prime }|\leq |t|\leq \frac{n}{2}$, and $|p|=|t|+|t^{\prime }|>\frac{n}{2}$. Hence, $|T^{t}|=n-|t|+1\geq \frac{n}{2}+1$. If $\frac{n}{4}>|T_{t}|=|t|$ then $\frac{n}{4}>|t^{\prime }|$. Thus, $|p|=|t|+|t^{\prime }|<\frac{n}{2}$, which is a contradiction. Thus, $|T_{t}|\geq \frac{n}{4}$. Finally, $|T^{t}|=n-|t|+1\leq n-\frac{n}{4}+1=\frac{3n}{4}+1$. ◻

A median vertex is usually non-unique, e.g., a rooted tree (a,(b,c)) has two median vertices *b* and *c*. Note that the concept of a median vertex is similar to the centroid of a tree, which partitions an unrooted tree into subtrees, each with a size of at most $\frac{n}{2}$. However, in our case, the median vertex divides a rooted binary tree into two subtrees, with the size of one subtree being between $\frac{n}{2}$ and $\frac{3n}{4}+1$, as shown in Lemma 1.

### Classification of taxon pairs

In the remaining part of the article, we assume that *T* and $T^{\prime }$ are two trees that share the same fixed set of leaves. Let *t* and $t^{\prime }$ represent fixed median vertices of *T* and $T^{\prime }$, respectively. Additionally, let ξ and $\xi^{\prime }$ denote the contribution functions of *T* and $T^{\prime }$, respectively. Without loss of generality, we further assume that all contribution functions ξ and $\xi^{\prime }$ are descending. That is, for any pair of vertices *v* and *w* such that v⪯w, it holds that ξ(v)≥ξ(w).

Given two trees *T* and $T^{\prime }$, we fix arbitrarily one median vertex in each tree. Then, the path connecting the median vertex with the root will be called a *median path*. We denote by *A* and *B* the sets of leaves excluding the median vertex in the upper and lower trees of *T*, respectively. Similarly, we denote $A^{\prime }$ and $B^{\prime }$ for $T^{\prime }$. Note that $|A|+|B|=|A^{\prime }|+|B^{\prime }|=n$.

We now have four possible classes for a pair of leaves ⟨x,y⟩, where *x* can be equal to *y*, from *T*, depending on their location:

- *AA* — if both leaves are located in the upper tree (i.e., x,y∈A),
- *BB* — if x,y∈B,
- *AB* — if x∈A and y∈B,
- *BA* — if x∈B and y∈A.

When considering the pair ⟨x,y⟩ in both trees, there are 16 possible *types* of locations, denoted in the form $XY|X^{\prime }Y^{\prime }$, where X,Y∈{A,B} and $X^{\prime },Y^{\prime }\in \{A^{\prime },B^{\prime }\}$. We say that the pair $\langle x,y\rangle \in L_{T}\times L_{T^{\prime }}$ has type $XY|X^{\prime }Y^{\prime }$ if $x\in X\cap X^{\prime }$ and $y\in Y\cap Y^{\prime }$.

Since some of the types due to symmetry represent the same sets of taxon pairs, we introduce categories for the joint representation of types as indicated in Table 1. There are 4 non-mixed categories $N_{1},N_{2},\ldots ,N_{4}$ that correspond uniquely to the types $AA|A^{\prime }A^{\prime }$, $AA|B^{\prime }B^{\prime }$, $BB|A^{\prime }A^{\prime }$ and $BB|B^{\prime }B^{\prime }$. Taking symmetry into account, there are 4 single-mixed categories $S_{1}\;-\;S_{4}$, e.g., in *S*_1_ the type $AB|A^{\prime }A^{\prime }$ is equivalent to the $BA|A^{\prime }A^{\prime }$ type, since they involve the same taxon pairs, and 2 double-mixed categories: *D*_1_ (which includes $AB|A^{\prime }B^{\prime }$ and $BA|B^{\prime }A^{\prime }$) and *D*_2_ (which includes $AB|B^{\prime }A^{\prime }$ and $BA|A^{\prime }B^{\prime }$).

**Table 1:** Categories of the types of taxon pair sets. The representative type of each category is marked by a star.

$XY|X^{\prime }Y^{\prime }$	$A^{\prime }A^{\prime }$	$A^{\prime }B^{\prime }$	$B^{\prime }A^{\prime }$	$B^{\prime }B^{\prime }$
*AA*	$N_{1}^{\;\;*}$	$S_{3}*$	*S*_3_	$N_{2}*$
*AB*	$S_{1}*$	$D_{1}*$	$D_{2}*$	$S_{2}*$
*BA*	*S*_1_	*D*_2_	*D*_1_	*S*_2_
*BB*	$N_{3}*$	$S_{4}*$	*S*_4_	$N_{4}*$

To compute $d_{p}(T,T^{\prime })$, it is sufficient to demonstrate how to compute it for one representative type $XY|X^{\prime }Y^{\prime }$ from each category, where X,Y∈{A,B} and $X^{\prime },Y^{\prime }\in \{A^{\prime },B^{\prime }\}$. Specifically, we calculate the *partial distances* as:


$$d_{XY|X^{\prime }Y^{\prime }}^{p}=\sum_{x\in X\cap X^{\prime }}\sum_{y\in Y\cap Y^{\prime }}{\left|\xi ({\text{lca}}_{T}(x,y))-\xi^{\prime }({\text{lca}}_{T^{\prime }}(x,y))\right|}^{p}.$$

Then, the cophenetic distance is the $\frac{1}{p}$-th power of the sum of the partial distances computed for each representative of the category. In the next sections, we show algorithms to compute partial distances for each representative type.

### The partial distance of non-mixed types

According to Table 1, there are four types of non-mixed taxon pairs: $AA|A^{\prime }A^{\prime }$, $AA|B^{\prime }B^{\prime }$, $BB|A^{\prime }A^{\prime }$, and $BB|B^{\prime }B^{\prime }$. To calculate the partial distance for each non-mixed type, we begin by contracting the trees *T* and $T^{\prime }$ to the set of leaves defined by the corresponding pair. For instance, for the type $BB|A^{\prime }A^{\prime }$, the trees *T* and $T^{\prime }$ are contracted to the set $B\cap A^{\prime }$.

We then compute the partial distance *d*_*p*_ for these contracted trees recursively. See also lines 14-15 in Algorithm 5.

If *f*(*n*) represents the complexity of the algorithm for computing the distance between trees of size *n*, the total time to calculate these four partial distances is $\sum_{i=1}^{4}f(c_{i})$, where $c_{1}=|A\;\cap \;A^{\prime }|$, $c_{2}=|A\;\cap \;B^{\prime }|$, $c_{3}=|B\;\cap \;A^{\prime }|$, and $c_{4}=|B\;\cap \;B^{\prime }|$ (note that $\sum_{i}c_{i}=n$.), plus the linear cost of performing the contractions.

### The partial distance of double-mixed types

Here, we present algorithms for computing partial distances for the categories *D*_1_ and *D*_2_, represented by the two double-mixed types $AB|A^{\prime }B^{\prime }$ and $AB|B^{\prime }A^{\prime }$, respectively.

#### Double-mixed types $AB|A^{\prime }B^{\prime }$.

The type $AB|A^{\prime }B^{\prime }$ denotes pairs ⟨x,y⟩, where *x* belongs to the upper trees and *y* to the lower trees of both *T* and $T^{\prime }$. In this case, the lca of ⟨x,y⟩ is positioned along the median path in both trees. Furthermore, ${\text{lca}}_{T}(x,y)$ is the same as ${\text{lca}}_{T}(x,t)$. Similarly, we have ${\text{lca}}_{T^{\prime }}(x,y)={\text{lca}}_{T^{\prime }}(x,t^{\prime })$.

Having this, the partial distance is

$$d_{AB|A^{\prime }B^{\prime }}^{p}=\sum_{x\in A\cap A^{\prime }}|\xi ({\text{lca}}_{T}(x,t))-\xi^{\prime }({\text{lca}}_{T^{\prime }}(x,t^{\prime })){|}^{p}\cdot |B\cap B^{\prime }|,\mathrm{\backslash vspace}*\mathrm{-5pt}$$ (1)

which can be computed in *O*(*pn*) time.

#### Double-mixed types $AB|B^{\prime }A^{\prime }$.

For $AB|B^{\prime }A^{\prime }$ the naïve approach requires $\Theta (pn^{2})$ steps. Below, we show an *O*(*pn*) time solution. We begin with the following problem.

**Problem 1.** *Given two sequences of numbers:* $\alpha_{1}\leq \alpha_{2}\leq \ldots \leq \alpha_{k}$ *and* $\beta_{1}\leq \beta_{2}\leq \ldots \leq \beta_{m}$. *Compute:* $\sum_{i,j}|\alpha_{i}-\beta_{j}{|}^{p}$.

**Algorithm 1 Function GetCntr (partially adapted from [24]).**

1: **Function** \fontsize7pt9pt\selectfontGetCntr(G,g,X), where *g* is a median vertex of *G*

2:     Set v.c:=0 for every vertex *v* on the median path of *G*

# *Init counters*

3:     **For** every leaf *l* in *X*: ${\mathrm{\backslash fontsize}7pt9pt\mathrm{\backslash selectfont}\text{lca}}_{G}(g,l).c={\mathrm{\backslash fontsize}7pt9pt\mathrm{\backslash selectfont}\text{lca}}_{G}(g,l).c+1$

4:     Initialize an empty sequence γ (a list)

5:     **For** every vertex *v* on the median path: append ξ(v) to

γ, repeated v.c times

6:     **Return** γ

**Lemma 2.** SeqPrd *from Algorithm 2 computes* $\sum_{i,j}|\alpha_{i}\;-\;\beta_{j}{|}^{p}$ *in* O(p(m+k)) *time and space.*

*Proof: Correctness:* Note that the swap in the 5-th line ensures that $\alpha_{k}\leq \beta_{m}$. Let $\lambda_{j}$ be the number of elements from α that are smaller or equal to $\beta_{j}$. In particular we have $\lambda_{m}=k$. The algorithm of SeqPrd in the main loop computes $\sigma_{j,l}$ as $\sum_{i=1}^{\lambda_{j}}\alpha_{i}^{l}$, i.e., it is the sum of the *l*-th powers of all elements from α that are smaller or equal to $\beta_{j}$. In particular, $\sigma_{m,l}=\sum_{i=1}^{k}\alpha_{i}^{l}$. Then, for a fixed *j*,

**Algorithm 2 Partial distances: type $AB|B^{\prime }A^{\prime }$.**

1: **Input:** *T* and $T^{\prime }$ with median vertices *t* and $t^{\prime }$, resp.

2: **Output:** $d_{AB|B^{\prime }A^{\prime }}^{p}(T,T^{\prime })$

3: **Function** $\mathrm{\backslash fontsize}7pt9pt\mathrm{\backslash selectfont}\text{SeqPrd}(p,\alpha_{1},\alpha_{2},\ldots ,\alpha_{k},\beta_{1},\beta_{2},\ldots ,\beta_{m})$:

4:     i:=j:=1

5:     **If** $\alpha_{k}>\beta_{m}$ **Then** swap α and β

6:     Let $\sigma_{j,l}:=0$ for all l∈{0,…,p} and j∈{0,…,m}

7:     **While** j≤m:

8:        **If** i≤k and $\alpha_{i}\leq \beta_{j}$

9:        **Then** *i* = *i* + 1; **For** $l\in \{0,\ldots ,p\}:\sigma_{j,l}=\sigma_{j,l}+\alpha_{i-1}^{l}$

10:        **Else** *j* = *j* + 1; **For** $l\in \{0,\ldots ,p\}:\sigma_{j,l}:=\sigma_{j-1,l}$

11:     **Return** ∑j=1m∑l=0p(pl)(−1) p−l(βjlσj,p−l+βjp−l(σm,l−σj,l))

12: Let $\alpha =\mathrm{\backslash fontsize}7pt9pt\mathrm{\backslash selectfont}\text{GetCntr}(T,t,A\cap B^{\prime })$ and $\beta =\mathrm{\backslash fontsize}7pt9pt\mathrm{\backslash selectfont}\text{GetCntr}(T^{\prime },t^{\prime },B\cap A^{\prime })$

13: **Return** \fontsize7pt9pt\selectfontSeqPrd(p,α,β)


∑i=1k|αi−βj|p=∑i=1λj(βj−αi)p+∑i=λj+1k(αi−βj)p=∑i=1λj∑l=0p(pl)βjl(−1)p−lαip−l+∑i=λj+1k∑l=0p(pl)αil(−1)p−lβjp−l=∑l=0p(pl)βjl(−1)p−l(∑i=1λjαip−l)+∑l=0p(pl)(−1)p−lβjp−l(∑i=λj+1kαil)=∑l=0p(pl)(−1)p−l(βjl∑i=1λjαip−l+βjp−l∑i=λj+1kαil).

Now, the formula in the 11-th line is derived by applying above the following identities $\sum_{i=1}^{\lambda_{j}}\alpha_{i}^{p-l}=\sigma_{j,p-l}$ and $\sum_{i=\lambda_{j}+1}^{k}\alpha_{i}^{l}=\sum_{i=1}^{k}\alpha_{i}^{l}-\sum_{i=1}^{\lambda_{j}}\alpha_{i}^{l}=\sigma_{m,l}-\sigma_{j,l}$.

*Time and space complexity:* The main loop requires O(m+k) steps, while computing the formula in the 11-th line requires *O*(*pm*) steps plus precomputing all values of binomial coeficients (pl) for l∈{0…p}, which can be done once in *O*(*p*) time. In total, the overall time complexity is O(p(m+k)), the same applies to space complexity. ◻

Now, $d_{AB|B^{\prime }A^{\prime }}^{p}$ and similarly, $d_{BA|A^{\prime }B^{\prime }}^{p}$ is computed by calling SeqPrd(p,α,β), where α is the sequence of contributions of ${\text{lca}}_{T}(x,t)$’s for *x* in $A\;\cap \;B^{\prime }$ and β is the sequence of contributions of ${\text{lca}}_{T^{\prime }}(y,t^{\prime })$’s for *y* in $B\cap A^{\prime }$. Such sequences are inferred in *O*(*n*) steps by the function GetCntr in Algorithm 1.

**Lemma 3.** *Algorithm 2 computes* $d_{AB|B^{\prime }A^{\prime }}^{p}$ *in O*(*pn*) *time.*

*Proof:* The proof is similar to the proof of Lemma 3 from [24]. The difference is in the more general computation of the sum from Lemma 2. We leave out simple details. ◻

### The partial distance of single-mixed types

There are four single-mixed categories represented by the types $AA|A^{\prime }B^{\prime }$, $AB|A^{\prime }A^{\prime }$, $BB|A^{\prime }B^{\prime }$, and $AB|B^{\prime }B^{\prime }$ (see Table 1). Each of these variants can be solved similarly. Thus, we present only the algorithm for computing the partial distance for $AA|A^{\prime }B^{\prime }$.

Assume that the pair of taxa ⟨x,y⟩ has the type $AA|A^{\prime }B^{\prime }$, meaning $x\in A\cap A^{\prime }$ and $y\in A\cap B^{\prime }$. In this case, ${\text{lca}}_{T}(x,y)$ is a vertex from the upper part of tree *T*, while ${\text{lca}}_{T^{\prime }}(x,y)$ lies on the median path of $T^{\prime }$. To apply our algorithm, we consider the tree to be ordered. For non-leaf vertices, the right child of a vertex *v* is denoted v.\textsf{rgh}, and the left child is denoted v.\textsf{lft}.

Let $x.\beta =\xi^{\prime }({\text{lca}}_{T^{\prime }}(x,t^{\prime }))\text{ for }x\in A\cap A^{\prime }$. Our solution is divided depending on the parity of *p*. We start with the solution to odd norms.

#### Single-mixed types under odd *L*_*p*_ norms.

We start with the following definitions.

-
$L_{v}^{+}:=\left\{ \begin{array}{cc} \emptyset & \text{if }v=\text{root}(T),\\ \left\{x:x\in A\cap A^{\prime }\cap L_{v}\text{ and }x.\beta \leq \xi (v.parent)\right\} & \text{otherwise}, \end{array}\right.$

- and $L_{v}^{-}:=(A\cap A^{\prime }\cap L_{v})⧵L_{v}^{+}$.

In the next two lemmas, we prove several properties of vertex attributes δ and σ from Algorithm 3.

**Algorithm 3 Partial distances: type $AA|A^{\prime }B^{\prime }$ for odd *p*’s.**

1: **Input:** *T* and $T^{\prime }$ with median verticest and $t^{\prime }$, respectively;   *p* is odd

2: **Output:** $d_{AA|A^{\prime }B^{\prime }}^{p}(T,T^{\prime })$

3: **For** every v in the upper tree of *T*:

4:     v.κ:=0; $v.\delta :=v.\sigma^{+}:=v.\sigma^{-}:=0\in {\mathbb{R}}^{p+1}$ # *the zero vector*

5: **For** $x\in A\cap A^{\prime }$: # *The preprocessing loop*

6:     $x.\beta =\xi^{\prime }({\mathrm{\backslash fontsize}7pt9pt\mathrm{\backslash selectfont}\text{lca}}_{T^{\prime }}(x,t^{\prime }))$; $\beta^{*}=\langle 1,x.\beta^{1},x.\beta^{2},\ldots ,x.\beta^{p}\rangle$

7:     **If** x.β≤ξ(x)

8:        **Then** $\omega_{x}:=\mathrm{\backslash arg}\min_{w}\{\xi (w)|x⪯w\text{\textbackslash{} and\textbackslash{} }x.\beta \leq \xi (w)\}$;

$\omega_{x}.\delta =\omega_{x}.\delta +\beta^{*}$

9:     **If** x.β≤ξ(x.\fontsize7pt9pt\selectfontparent) **Then** $x.\sigma^{+}:=\beta^{*}$ **Else** $x.\sigma^{-}:=\beta^{*}$

10: **For** every non-root v in the upper tree of *T* in postfix

order: # *The main loop*

11:     $v.\sigma^{+}:=v.\mathrm{\backslash fontsize}7pt9pt\mathrm{\backslash selectfont}\text{lft}.\sigma^{+}+v.\mathrm{\backslash fontsize}7pt9pt\mathrm{\backslash selectfont}\text{rgh}.\sigma^{+}-v.\delta$;

$v.\sigma^{-}:=v.\mathrm{\backslash fontsize}7pt9pt\mathrm{\backslash selectfont}\text{lft}.\sigma^{-}+v.\mathrm{\backslash fontsize}7pt9pt\mathrm{\backslash selectfont}\text{rgh}.\sigma^{-}+v.\delta$

12:     $Y_{v}=A\cap B^{\prime }\cap L(v.\mathrm{\backslash fontsize}7pt9pt\mathrm{\backslash selectfont}\text{sibling})$

13:     v.κ:=|Yv|∑l=0p(pl)(−1) p−l(ξ(v.\fontsize7pt9pt\selectfontparent) lv.σp−l++ξ(v.\fontsize7pt9pt\selectfontparent) p−lv.σl−)

14: **Return** $\sum_{v\in T}v.\kappa$

**Lemma 4.** *If v is strictly internal, then after the preprocessing loop of Algorithm 3, we have*


$$v.\delta =\langle \sum_{x\in \Omega_{v}}(x.\beta {)}^{l}:l\in \{0,1,\ldots ,p\}\rangle ,$$

*where* $\Omega_{v}=\{x\in L_{v}\cap A\cap A^{\prime }:\xi (v)\geq x.\beta >\xi (v.\text{parent})\}$.

*Proof:* In line 8 of Algorithm 2, $\omega_{x}$ is defined as the vertex such that $x.\beta \leq \xi (\omega_{x})$, and this inequality is not satisfied by the parent of $\omega_{x}$; that is, $x.\beta >\xi (\omega_{x}.\text{parent})$. Combining these observations, we can conclude that $\omega_{v}$ consists of vertices $x\in A\;\cap \;A^{\prime }$ for which $v=\omega_{x}$ as specified in line 8. Consequently, from the assignment in line 8, we have

$v.\delta =\langle \sum_{x\in \Omega_{v}}(x.\beta ){\;}^{l}:l\in \{0,1,\ldots ,p\}\rangle$. ◻

**Lemma 5.** *If v is strictly internal, then after the main loop of Algorithm 3,*

- $v.\sigma^{+}=\langle \sum_{x\in L_{v}^{+}}x.\beta^{l}:l\in \{0,1,\ldots ,p\}\rangle$,
- and $v.\sigma^{-}=\langle \sum_{x\in L_{v}^{-}}x.\beta^{l}:l\in \{0,1,\ldots ,p\}\rangle$.

*Proof:* We present an inductive proof for a fixed l∈{0,1,…,p} and for $v.\sigma_{j+1}^{+}$, which denotes the (j+1)-th element of the vector $v.\sigma^{+}$. If *v* is a leaf, then the equality follows directly from line 9 of Algorithm 3 and the definition of $L_{v}^{+}$. Now, let us consider the case where *v* is strictly internal. Note that ξ(v)≥ξ(v.parent).


$$v.\sigma_{l+1}^{+}=v.\text{\textbackslash{}textsf\{lft}\}.\sigma_{l+1}^{+}+v.\text{\textbackslash{}textsf\{rgh}\}.\sigma_{l+1}^{+}-v.\delta_{l+1}$$

$$=\sum_{x\in L_{v.\text{\textbackslash{}textsf\{lft}}^{+}}\}x.\beta^{l}+\sum_{x\in L_{v.\text{\textbackslash{}textsf\{rgh}}^{+}}\}x.\beta^{l}-v.\delta_{l+1}$$

=∑x∈A∩A′∩Lv.\textsf{lftx.β≤ξ(v.\textsf{lft.parent)}}x.βl+∑x∈A∩A′∩Lv.\textsf{rghx.β≤ξ(v.\textsf{rgh.parent)}}x.βl−v.δl+1

=∑x∈A∩A′∩Lvx.β≤ξ(v)x.βl−∑x∈A∩A′∩Lvξ(v)≥x.β>ξ(v.parent)x.βl

=∑x∈A∩A′∩Lvx.β≤ξ(v.parent)x.βl=∑x∈Lv+x.βl.

The proof for $v.\sigma^{-}$ is similar. We omit details. ◻

**Lemma 6.** *If v is strictly internal in T*, *then after the main loop of Algorithm 3 we have*

$$v.\kappa =\sum_{x\in A\cap A^{\prime }\cap L_{v}}\sum_{y\in A\cap B^{\prime }\cap L(v.\text{\textbackslash{}textsf\{sibling}})\}|\xi ({\text{lca}}_{T}(x,y))-\xi^{\prime }({\text{lca}}_{T^{\prime }}(x,y){|}^{p}.$$ (2)

*Proof:* We can prove (2) by using Lemmas 4 and 5. Let *v* be strictly internal in the upper tree of *T*. Then, for a leaf $x\in A\cap A^{\prime }\cap L_{v}$ and a leaf *y* from $Y_{v}$ (i.e., from $A\cap B^{\prime }\cap L(v.\text{\textbackslash{}textsf\{sibling}\})$, see line 12), we have ${\text{lca}}_{T}(x,y)=v.\text{parent}$ and $\xi^{\prime }({\text{lca}}_{T^{\prime }}(x,y))=\xi^{\prime }({\text{lca}}_{T^{\prime }}(x,t^{\prime }))=x.\beta$. Let *R* be the right side of (2). Then,


$$R=\sum_{x\in A\cap A^{\prime }\cap L_{v}}\sum_{y\in Y_{v}}|\xi (v.\text{parent})-x.\beta {|}^{p}$$

=∑y∈Y(∑x∈A∩A′∩Lvx.β≤ξ(v.parent)(ξ(v.parent)−x.β)p−∑x∈A∩A′∩Lvx.β>ξ(v.parent)(ξ(v.parent)−x.β)p)

$$=|Y_{v}|(\sum_{x\in L_{v}^{+}}(\xi (v.\text{parent})-x.\beta {)}^{p}-\sum_{x\in L_{v}^{-}}(\xi (v.\text{parent})-x.\beta {)}^{p})$$

=|Yv|∑l=0p(pl)(−1)p−l(ξ(v.parent)lv.σp−l+1+−ξ(v.parent)p−lv.σl+1−)

The last equation is obtained by identities from Lemma 5 and binomial expansions. *R* equals the right side of the assignment from line 13. ◻

**Lemma 7.** *Algorithm 3 computes* $d_{AA|A^{\prime }B^{\prime }}^{p}$ in O(nlogn+pn) *time.*

*Proof:* We show the algorithm’s correctness followed by the stated time complexity.

*Correctness:* Let *I* be the set of non-root vertices from the upper tree of *T*. Then, every pair of leaves ⟨x,y⟩ of type $AA|A^{\prime }B^{\prime }$ uniquely determines v∈I such that ${\text{lca}}_{T}(x,y)=v.\text{parent}$ and $x\in L_{v}$. It also follows that y∈L(v.\textsf{sibling}). Let x⊕y denote such vertex *v*. For a given *v*, $A\cap A^{\prime }\cap L_{v}\times A\cap B^{\prime }\cap L(v.\text{\textbackslash{}textsf\{sibling}\})$ is the set of all pairs ⟨x,y⟩ such that x⊕y=v. Hence,


$$d_{AA|B^{\prime }A^{\prime }}^{p}=\sum_{v\in I}\sum_{x\in A\cap A^{\prime }\cap L_{v}}\sum_{y\in A\cap B^{\prime }\cap L(v.\text{\textbackslash{}textsf\{sibling}})\}|\xi ({\text{lca}}_{T}(x,y))-\xi^{\prime }({\text{lca}}_{T^{\prime }}(x,y)){|}^{p}.$$

By Lemma 6 the above sum equals the value returned in the last line of Algorithm 3.

*Time complexity:* The key aspect of the time complexity is found in line 8. We show that ω can be found by a binary search in O(logn) time that seeks the value in an ordered array composed of vertices on the path connecting a given leaf *x* with the root of *T*. An infix traversal of *T* can construct such an array. Then, a vertex is inserted into the array when visited for the first time. When a vertex is visited for the last time, it is removed from the array. Due to the monotonic ordering of paths, the array is always sorted, and its size is limited by *n*. The time complexity of the remaining loops is *O*(*pn*), which gives O(nlogn+pn) time of Algorithm 3. ◻

#### Single-mixed types under even *L*_*p*_ norms.

**Algorithm 4 Partial distances: type $AA|A^{\prime }B^{\prime }$ for even *p*.**

1: **Input:** *T* and $T^{\prime }$ with median vertices *t* and $t^{\prime }$, resp.

2: **Output:** $d_{AA|A^{\prime }B^{\prime }}^{p}(T,T^{\prime })$

3: **For** every *v* in the upper tree of *T*: v.κ:=0. $v.\sigma :=0\in R^{p+1}$

(the zero vector)

4: **For** $x\in A\cap A^{\prime }$: # *Preprocessing*

5:     $x.\beta =\xi^{\prime }({\mathrm{\backslash fontsize}7pt9pt\mathrm{\backslash selectfont}\text{lca}}_{T^{\prime }}(x,t^{\prime }));$ $x.\sigma :=\langle x.\beta^{l}:l\in \{0,1,\ldots ,p\}\rangle$

6: **For** every non-root *v* in the upper tree of *T* in postfix

order:

7:     v.σ:=v.\fontsize7pt9pt\selectfontlft.σ+v.\fontsize7pt9pt\selectfontrgh.σ

8:     v.κ:=|A∩B′∩L(v.\fontsize7pt9pt\selectfontsibling)|·∑l=0p(pl)(−1) p−lξ(v.\fontsize7pt9pt\selectfont\textsf{parent}) p−lv.σl

9: **Return** $\sum_{v\in T}v.\kappa$

The advantage of even *L*_*p*_ norms is given by the relation $(|a-b|){\;}^{p}=(a-b){\;}^{p}$ for *p* even. This fact allows us to circumvent the extra complexity of Algorithm 3. Algorithm 4 outlines the computation of $d_{AA|A^{\prime }B^{\prime }}^{p}$ when *p* is even.

**Lemma 8.** *For each non-root vertex v in the upper tree of T after Algorithm 4 we have*

- $v.\sigma =\langle \sum_{x\in A\cap A^{\prime }\cap L_{v}}(x.\beta ){\;}^{l}:l\in 0,\ldots ,p\rangle$,
- $v.\kappa =\sum_{x\in A\cap A^{\prime }\cap L_{v}}\sum_{y\in A\cap B^{\prime }\cap L(v.\text{\textbackslash{}textsf\{sibling}})\}|\xi ({\text{lca}}_{T}(x,y))-\xi^{\prime }({\text{lca}}_{T^{\prime }}(x,y)){|}^{p}$.

*Proof:* The relation for v.σ follows directly from the bottom-up nature of the algorithm and the relation for v.κ can be observed using the following equation:


∑x∈A∩A′∩Lv∑y∈A∩B′∩L(v.\textsf{sibling)}|ξ(lcaT(x,y))−ξ′(lcaT′(x,y))|p=∑x∈A∩A′∩Lv∑y∈A∩B′∩L(v.\textsf{sibling)}(x.β−ξ(v.parent))p=|A∩B′∩L(v.\textsf{sibling})|∑l=0p(pl)(−1)p−lξ(v.\textsf{parent})p−l∑x∈A∩A′∩Lv(x.β)l.

◻

**Lemma 9.** *Algorithm 4 computes* $d_{AA|A^{\prime }B^{\prime }}^{p}(T,T^{\prime })$ *in O*(*pn*) *time for even p*.

*Proof:* The correctness of the algorithm follows from Lemmas 8 and 7. Finally, the time complexity of Algorithm 4 is *O*(*pn*) since each line within the loops requires *O*(*p*) time and is executed at most 2*n* times. ◻

#### Partial distances of the remaining single-mixed types.

As previously mentioned, Algorithm 3 and Algorithm 4 for computing the partial distances of single mixed type $AA|A^{\prime }B^{\prime }$ can be adapted to solve the other types as follows. For the type $BB|A^{\prime }B^{\prime }$, replace the term “upper” with “lower” and *A* with *B* in both algorithms. For the type $AB|A^{\prime }A^{\prime }$, swap the input trees and execute both algorithms. For the type $AB|B^{\prime }B^{\prime }$, swap the input trees and run the algorithm designed for the type $BB|A^{\prime }B^{\prime }$. These modifications are detailed in lines 14 through 16 of Algorithm 5.

### Algorithm to compute the cophenetic distance under the *L*_*p*_ norm

The pseudo-code in Algorithm 5 summarizes the complete procedure for computing the cophenetic distance for finite *p*. The correctness of the algorithm follows from the results presented in the previous section. Below, we analyze the time complexity in two scenarios: when *p* is constant, and when *p* is considered as a parameter in the asymptotic analysis.

**Algorithm 5 Computing cophenetic distance.**

1: **Input:** *T* and $T^{\prime }$ with the same set of leaves of size *n*, two

ascending contribution functions $\xi :V_{T}\to R$ and $\xi^{\prime }:V_{T^{\prime }}\to R$,

and an integer p≥1

2: **Output:** The *L*_*p*_-cophenetic distance between *T* and $T^{\prime }$ with

respect to ξ and $\xi^{\prime }$

3: Compute all binomial coeficients ai=(pi), for all *i*, by

$a_{i}=\frac{p-i+1}{i}a_{i-1}$ and *a*_0_ = 1

4: **Function** $\mathrm{\backslash fontsize}7pt9pt\mathrm{\backslash selectfont}\text{PartDist}(T,T^{\prime })$

5:     Compute *t* and $t^{\prime }$ the median vertices of *T* and $T^{\prime }$,

resp.

6:     Let *A* and $A^{\prime }$ be the set of all leaves in the upper

tree of *T* and $T^{\prime }$, resp.

7:     s:=0; $B:=L_{T}⧵A$; $B^{\prime }:=L_{T^{\prime }}⧵B$

8:     For *X*, in {A,B}: For $X^{\prime }$ in $\left\{A^{\prime },B^{\prime }\right\}$

9:        Let $S:=\mathrm{\backslash fontsize}7pt9pt\mathrm{\backslash selectfont}\text{contract}(T,X\cap X^{\prime })$ and $S^{\prime }:=\mathrm{\backslash fontsize}7pt9pt\mathrm{\backslash selectfont}\text{contract}(T^{\prime },X\cap X^{\prime })$

10:        $s=s\;+\;\mathrm{\backslash fontsize}7pt9pt\mathrm{\backslash selectfont}\text{PartDist}(S,S^{\prime })$ # *Compute partial distances*

*for type* $XX|X^{\prime }X^{\prime }$

11:     $s=s+d_{AB|A^{\prime }B^{\prime }}^{p}$ see Eq. (1) # *Double-mixed $AB|A^{\prime }B^{\prime }$*

12:     $s=s+d_{AB|B^{\prime }A^{\prime }}^{p}$ by Alg. 2 # *Double-mixed $AB|B^{\prime }A^{\prime }$*

13:     # *All single-mixed types*

14:     $s=s+d_{AA|A^{\prime }B^{\prime }}^{p}$ using Alg. 3 if *p* is odd, or Alg. 4

otherwise

15:     Similarly to line 14 , $s=s\;+\;d_{BB|A^{\prime }B^{\prime }}^{p}$, but replace

“upper” with “lower” and *A*

with *B* in Alg. 3/4

16:     For $AB|A^{\prime }A^{\prime }$ and $AB|B^{\prime }B^{\prime }$ swap the input trees and

repeat lines 14 and 15

17:     **Return** *s* # *Return the sum of partial distances*

18: **Return** $\mathrm{\backslash fontsize}7pt9pt\mathrm{\backslash selectfont}\text{PartDist}(T,T^{\prime }){\;}^{\frac{1}{p}}$ # *Return the cophenetic distance*

First, consider the case where *p* is constant. Since pn+nlogn∈O(nlogn), it follows from Lemmas 3 and 7 that computing the partial distances of mixed types requires O(nlogn) time when *p* is odd, and *O*(*n*) time when *p* is even. Consequently, the overall time complexity of Algorithm 5, as derived in [24], is $O(n\log^{2}\;n)$ for odd *p*, and O(nlogn) for even *p*.

If *p* is a parameter, f(n,p) is the worst-case time complexity of the complete algorithm. Then, computation of partial distances of non-mixed types (see lines 8-10 in Algoritm 5) requires $f(c_{1},p)+f(c_{2},p)+f(c_{3},p)+f(c_{4},p)+O(n)$ time where $\sum c_{i}=n$ and $0\leq c_{i}\leq \mathrm{.75}n+1$ for each *i* (by Lemma 1), while the computation of mixed types requires O(nlogn+pn) time if *p* is odd or *O*(*pn*) time if *p* is even, (Lemmas 3 and 7). Therefore, for some k≥1 we can write that, f(n,p)=1 if n≤5, $f(n,p)=k(n\log n\;+\;pn)\;+\;\max_{0\leq c_{i}\leq \mathrm{.75}n+1}f(c_{1},p)\;+\;f(c_{2},p)\;+\;f(c_{3},p)$ $+\;f(c_{4},p)$, if *n*>5 and *p* is odd, and $f(n,p)=kpn\;+\;\max_{0\leq c_{i}\leq \mathrm{.75}n+1}f(c_{1},p)\;+\;f(c_{2},p)\;+\;f(c_{3},p)$ $+\;f(c_{4},p)$, otherwise.

**Theorem 10.** *The time complexity of the algorithm is* $O(n\log^{2}\;n\;+\;pn\log n)$, *when p is odd and* O(pnlogn) *when p is even.*

*Proof: The proof for odd norms*. Assume that *p* is odd. We show that there are constants b≥1 and *d*>0 such that for every *n*>0 and *p*>0, $f(n,p)\leq d(n\log^{2}\;n\;+\;pn\log n)+b$. The proof is by induction on *n*. For n≤5 we have f(n,p)=1 and the inequality is satisfied. For *n*>5, $f(n,p)=kn\log n+kpn+\max\sum_{i}f(c_{i})\leq kn\log n+kpn+4b+d\max\sum_{i}(c_{i}\log^{2}c_{i}+pc_{i}\log c_{i})\leq kn\log n+kpn+4b+dn\log^{2}(\mathrm{.75}n+1)+dpn\log(\mathrm{.75}n+1)$.

Let $d=-bk/\log\frac{11}{12}\approx bk7.966$. Then, for *n*>5, $\log(\mathrm{.75}\;+\;\frac{1}{n})\leq \log(\mathrm{.75}\;+\;\frac{1}{6})=\log\frac{11}{12}$. Also, $d(\log(\mathrm{.75}n\;+\;1)\;-\;\log n)=d\log(\mathrm{.75}\;+\;\frac{1}{n})\leq d\log\frac{11}{12}=-bk$ and $d(\log^{2}(\mathrm{.75}n\;+\;1)\;-\;\log^{2}\;n)$$=\;d\log(\mathrm{.75}\;+\;\frac{1}{n})\log((\mathrm{.75}n\;+\;1)n)\leq \;-\;bk\log((\mathrm{.75}n\;+\;1)n)\leq -bk\log n\;-\;bk$. Finally, for *n*>5, $f(n)-dn\log^{2}\;n-dnp\log n-b\leq kn\log n\;+\;3b\;+\;dn(\log^{2}(\mathrm{.75}n\;+\;1)\;-\;\log^{2}\;n)\;+\;dpn(\log(\mathrm{.75}n\;+\;1)-\log n)\leq (1-b)kn\log n+3b-nbk+pn(-bk)<0$.

*Even norms*. If *p* is even, it suffices to show that f(n,p)≤dpnlogn+b, for some constants *d*>0 and b≥1. The proof is similar and simpler compared to the odd case and follows the same reasoning. We omit details. ◻

The last theorem shows that, despite the higher complexity of odd norms, the factor *p* only appears in the asymptotically minor term of $O(n\log^{2}\;n\;+\;pn\log n)$. This suggests that the computational effort for cophenetic distances is more influenced by the size of the input trees than by the chosen norm level *p*. Additionally, this theoretical result highlights that the binary search algorithm - required for computing double-mixed types and representing the main distinction between the two algorithmic variants - introduces $O(n\log^{2}\;n)$ more steps in the worst-case than the algorithm for even norms. See the experimental section for a detailed discussion on scalability and a comparison of the algorithms for odd and even norms.

#### Cophenetic distances under $L_{\infty }$ norm.

Algorithm 5 can be easily adapted to solve the last remaining case of cophenetic distances. The algorithm for the $L_{\infty }$ norm is similar to the case *p* = 1. The only difference is taking the maximum value instead of adding partial distances as follows. First, fix p:=1. Then in line 7, replace s:=0 with s:=−∞. In lines 10-16 instead of assignments *s* = *s* + *r*, where *r* is the right-hand expression, write s:=max{s,r}. Now, the time complexity is $O(n\log^{2}\;n)$, which follows from Theorem 10 for the odd case *p* = 1.

## Results

In the following sections, we present the scalability study and distribution analysis results. The near-linear time algorithm, i.e., Algorithm 5, for computing the *L*_*p*_ norm cophenetic distance and the naïve (quadratic time) algorithm were implemented in Rust 1.79.0. The implementation of both algorithms, along with instructions on reproducing the simulation results below, is available on GitHub [25]. Note that the distances computed by the naïve and our algorithm are the same.

### Scalability analysis

We investigate the scalability of our algorithm by comparing its runtime to that of the best-known quadratic-time algorithm for computing cophenetic distance.

**Fig 2:**
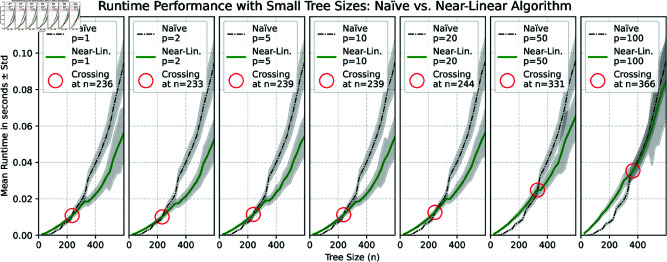
Scalability analysis comparing the near-linear algorithm and the naïve quadratic-time algorithm. The diagrams show the average runtime with standard deviation bands over 1000 runs for each tree size (n=25,50,…,600) on pairs of random trees, evaluated for *L*_*p*_-norms (p=1,2,5,10,20,50,100). Crossing points indicate the tree sizes where the near-linear algorithm outperforms the naïve one.

**Fig 3:**
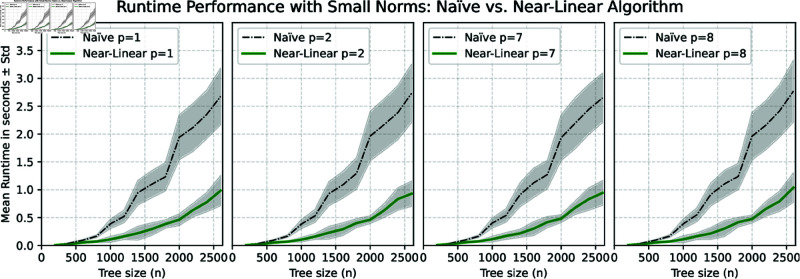
Scalability analysis comparing the near-linear algorithm and the naïve quadratic-time algorithm for small norms. The diagrams show the average runtime with standard deviation bands over 1,000 runs for each tree size (n=200,400,…,2600) on pairs of random trees, evaluated for *L*_*p*_-norms (p=1,2,7,8).

**Fig 4:**
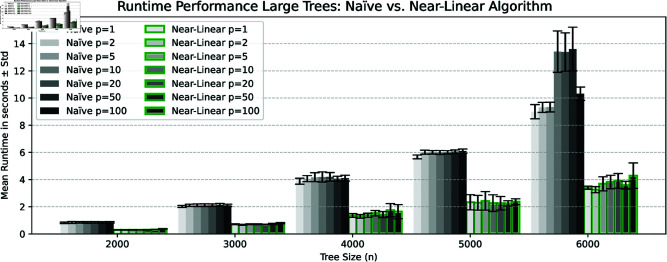
Scalability analysis comparing the near-linear algorithm (outlined bars) and the naïve quadratic-time algorithm on large trees. The diagrams show the average runtime with standard deviation error over 100 runs for each tree size (n=1000,2000,…,6000) on pairs of random trees, evaluated for *L*_*p*_-norms (p=1,2,5,10,20,50,100).

**Datasets.** We generated three datasets consisting of pairs of random binary trees with *n* leaves using the classic Yule model, where for each *n* we generated *q* pairs. The first dataset was generated using n=25,50,…,600 and *q* = 1000, the second with n=200,400,…,2600 and *q* = 1000, and the last one with n=1000,2000,…,6000 and *q* = 100. Algorithms were executed on each pair of trees for various norms *L*_*p*_ with *p* values ranging from 1 to 100. The comparative runtime analysis for each dataset is illustrated in the diagrams of Figs 2, 3, and 4.

**Results.** Our algorithm shows a significant improvement in efficiency compared to the quadratic solution for trees with more than 250 taxa and norms below 20, as illustrated in Fig 2. Furthermore, the crossing point at which our algorithm outperforms the quadratic solution increases gradually as *p* increases, reaching 366 at *p* = 100. This finding indicates the potential for developing a hybrid algorithm that utilizes the divide-and-conquer method for larger trees while reverting to the quadratic time algorithm for trees with fewer than 230–370 leaves during recursion.

Despite the differences in asymptotic worst-case time complexity between odd and even norms, these differences are not evident in the average runtime diagrams presented in Figs 3 and 4. Many of these differences can be attributed to the binary search step in Algorithm 3, as explained in Lemma 7, where the runtime is relatively straightforward to estimate. Notably, the computation of odd norms accounted for only 0.01% of the total average runtime in the binary search steps. In practical terms, this indicates that the time complexity of Algorithm 3 is closer to *O*(*pn*) rather than the more conservative estimate of O(nlogn+pn) provided in Lemma 7. Consequently, the overall runtime complexity for calculating the cophenetic distance can be approximated as O(pnlogn) for any given norm. Since the trees were generated randomly, we propose that O(pnlogn) is also a valid estimator of the average time complexity of our algorithm. However, as shown in Fig 4, there are no significant differences in runtime across various norms for a fixed tree size *n*. This indicates that the runtime is primarily affected by the overhead associated with maintaining supporting data structures, rather than by the computation of values, which involves loops over the range from 0 to *p*. We conjecture that for extremely large values of *p*, the computational cost of these loops will become increasingly noticeable in the runtime; however, such scenarios were not tested in this study.

### Cophenetic distance distributions

We investigate the distributions of three key representatives from the generalized class of cophenetic distances. These representatives are (i) the original cophenetic distance metric defined by depths, (ii) the metric defined by the heights of the subtrees, and (iii) the metric defined by the number of taxa in the subtrees. For ease of reference, we refer to these metrics as the *depth*, *subtree-height*, and *subtree-size* cophenetic metrics, respectively.

To the best of our knowledge, the specific distributions for any selected metrics remain unpublished. Therefore, we present here the sampled distributions of cophenetic distances based on two standard models of phylogenetic tree sampling: the uniform model and the Yule model [28,29]. The sampled distributions for the classical depth-based cophenetic distance were previously discussed in [1] for both the *L*_1_ norm and the *L*_2_ norm under the uniform model.

**Data.** We generated a dataset containing 10^6^ pairs of trees, each with 100 leaves. Each tree was independently generated under the uniform model. Similarly, we generated another dataset based on the Yule model. It is important to note that the generated trees do not include edge lengths; therefore, both depths and subtree heights are measured in terms of the number of edges.

**Fig 5:**
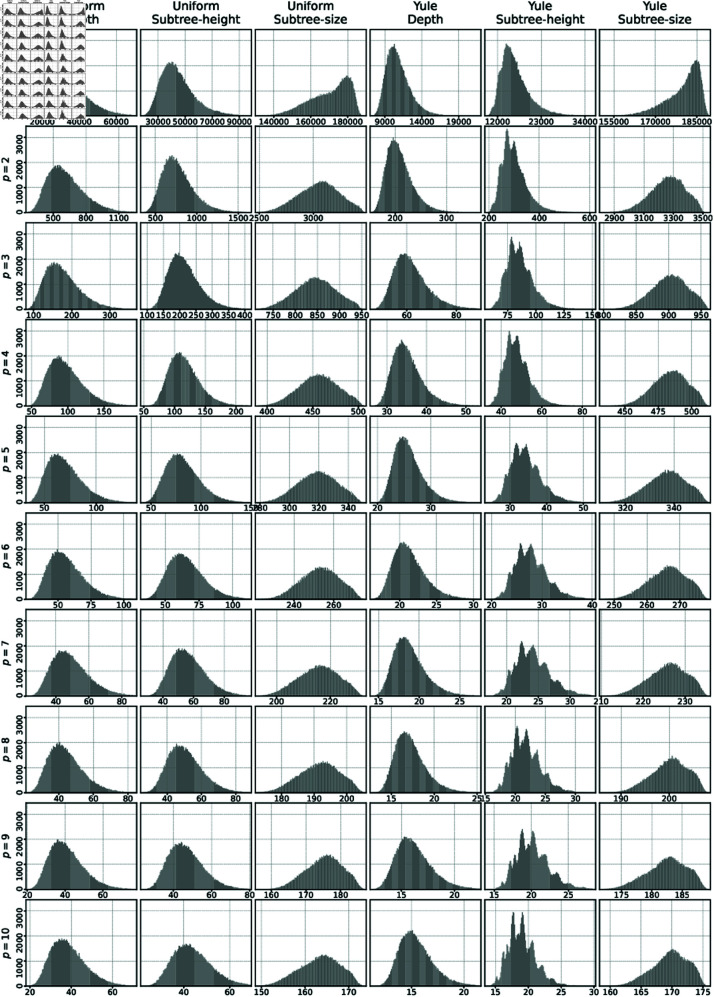
Sampled distribution of three cophenetic distances under the *L*_*p*_ norms for *p* = 1 to 10 (rows). Pairs of trees of size 100 were sampled according to the uniform model (left columns) and the Yule model (right columns). The frequencies are grouped into 200 bins. Note that the y-axis scale and range are the same across all diagrams. To enhance visibility, low frequencies (e.g., at low distances close to 0) are omitted and the width of the diagrams is appropriately adjusted.

**Fig 6:**
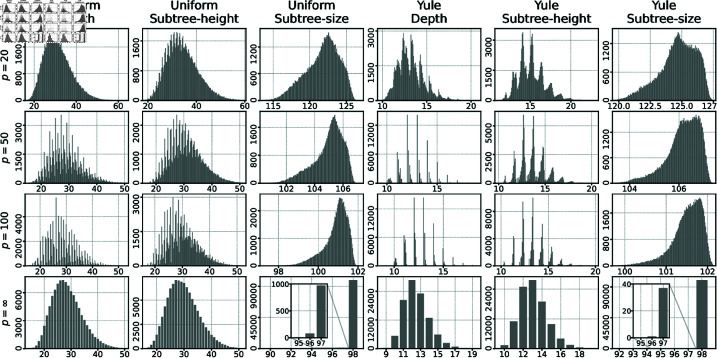
(Cont. from Fig 5). Sampled distribution of three cophenetic distances is shown for *L*_*p*_ norms with *p* = 20, 50, 100, and ∞ (rows). Similar to Fig 5, the frequencies for finite *p* are grouped into 200 bins. Since all three contribution functions return integers, the $L_{\infty }$ norms are also integers. As a result, the frequency values for $L_{\infty }$ are presented without binning.

**Fig 7:**
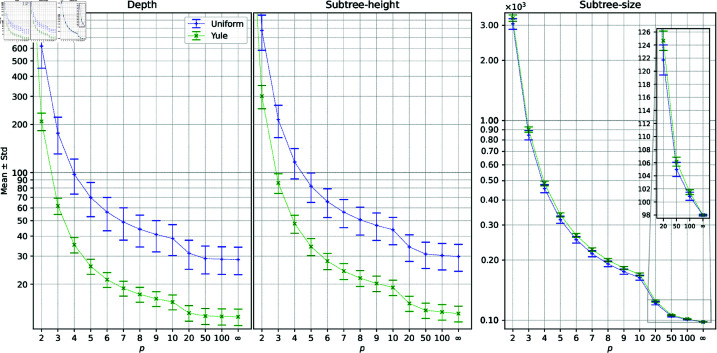
Mean and standard deviation from the sampled distributions for the uniform and Yule models for *L*_*p*_ norms with *p*>1.

**Results.** The sampled distributions are illustrated in Figs 5 and 6, with the corresponding mean and standard deviation statistics depicted in Fig 7.

The distribution of subtree-height and depth cophenetic metrics exhibit similar shapes under the uniform model for smaller values of *p* as illustrated in Fig 5. A similar trend is observed for the depth under the Yule model. Furthermore, these distributions are positively skewed, which is consistent with the findings for the *L*_1_ and *L*_2_ norms of the depth cophenetic metric reported in [1]. On the contrary, the subtree-height distributions exhibit positive skewness. It is also worth noting that our study involved a significantly larger number of pairs for sampling. As a result, the diagrams in Fig 5 appear smoother compared to the sampled distributions of depth metric from [1].

Since cophenetic vectors are finite, it is straightforward to prove that $\lim_{p\to \infty }d_{p}(T,T^{\prime })=d_{\infty }(T,T^{\prime })$ under any contribution function. Consequently, *L*_*p*_ cophenetic distributions converge to $L_{\infty }$ distributions as *p* tends to infinity. Furthermore, in our case, all three contribution functions return integer values, which implies that the $L_{\infty }$ distributions have an integer domain, as illustrated in Fig 6. This property is evident in diagrams, where the distributions become increasingly irregular as *p* grows and more and more similar to the corresponding discrete distributions under $L_{\infty }$. This effect is particularly noticeable for the depth and subtree-height metrics. By contrast, the distributions for the subtree-size metric remain relatively smooth. This smoothness arises from the fact that, under the $L_{\infty }$ norm, almost all frequencies under uniform and Yule models are concentrated at 98 (see low standard deviation in Fig 7). This value is derived from the pair of leaf labels that form a cherry in one tree but are separated by the root in the other tree, yielding subtree sizes of 2 and 100, respectively, and a corresponding distance of 98.

The sampled distributions for the subtree-size cophenetic metric are notably distinct from the analysis mentioned above. The histograms for this metric are *negatively*-skewed, and the mean value under the Yule model is, in this case, larger than the mean value under the uniform model.

In comparing the depth and subtree-height cophenetic metrics, we find that the mean values under the Yule model are significantly lower than those under the uniform model. A similar bias towards the Yule model was previously noted in the sampled distributions for the path-difference distance [30,31].

Additionally, the mean and standard deviation of the depth metric under the *L*_1_ and *L*_2_ norms align with the exact values reported in [32].

## Results and discussion

We introduced a novel algorithmic framework for computing the *L*_*p*_ norm cophenetic distance, achieving a time complexity of $O(n\log^{2}\;n\;+\;pn\log n)$ for odd values of *p*, O(pnlogn) for even values of *p*, and O(nlogn) for $L_{\infty }$ norm. This represents a substantial advancement compared to the previously best-known naïve algorithm, which requires $\Theta (pn^{2})$ time.

Additionally, our scalability studies suggest that the estimated runtime of our algorithm approaches O(pnlogn) time under all *L*_*p*_ norms with finite *p*, contrasting with the larger upper asymptotic bound observed for odd values of *p*. These advancements greatly improve the usability of the cophenetic distance for large-scale phylogenetic studies and the median-tree inference of species trees from gene trees using this metric.

Distribution analyses of these three key representative metrics from the cophenetic class further enhance this work, offering practitioners valuable guidance in selecting appropriate metrics for their specific needs.

The framework demonstrates broad practical applicability by generalizing to all metrics that rely on the path-monotonic property, here referred to as the class of *generalized cophenetic distances*. This generalization can be achieved by either designing contribution functions that satisfy basic monotonicity properties or by forming linear, positively weighted combinations of existing contribution functions.

As a result, the class of generalized cophenetic distances includes the original cophenetic distance based on depth, as well as other metrics, particularly the subtree height and subtree size cophenetic distances. Additionally, this class incorporates the more recently proposed metric from [19], which combines both weighted and unweighted contribution functions. Consequently, this metric can be computed in near-linear time using our algorithm under any *L*_*p*_ norm.

Furthermore, the framework can be applied to mixed scenarios where different types of cophenetic vectors are used for the trees — for example, depth in one tree and height in another. Although these mixed scenarios may not fully satisfy metric properties, they can still be useful for comparing trees from different origins by allowing asymmetry. For instance, this approach may be more suitable when comparing a gene tree, which represents the evolutionary history of a gene, to a species tree that illustrates the evolutionary history of the species from which the genes were sampled.
